# Room-Temperature Raman Enhancement in Colloidal 2D PbS Nanosheets

**DOI:** 10.1021/acs.jpclett.6c02525

**Published:** 2026-09-02

**Authors:** Sabin Aryal, Yiteng Tang, Liangfeng Sun

**Affiliations:** † Department of Physics and Astronomy, 1888 Bowling Green State University, Bowling Green, Ohio 43403, United States; ‡ Center for Photochemical Sciences, 1888 Bowling Green State University, Bowling Green, Ohio 43403, United States

## Abstract

Colloidal two-dimensional (2D) PbS nanosheets exhibit a dramatically enhanced longitudinal optical (LO) phonon Raman signal at room temperature, exceeding the intensity of 0D quantum dots by orders of magnitude. We attribute this giant enhancement to two synergistic mechanisms: spatial phonon coherence across the expansive 2D lattice and broad resonance enhancement enabled by a step-like density of states that strengthens exciton–phonon coupling. While surface oxidation can activate room-temperature Raman scattering in 0D quantum dots, their signal remains significantly weaker than that of pristine 2D nanosheets. Structural coalescence from porous quantum-dot networks to continuous 2D nanosheets directly drives peak sharpening and intensity amplification. Unlocking this robust, non-destructive Raman response provides a key framework to investigate 2D lattice dynamics, which govern hot-carrier relaxation, multiple exciton generation, and superfluorescence in colloidal quantum materials.

## Graphical abstract

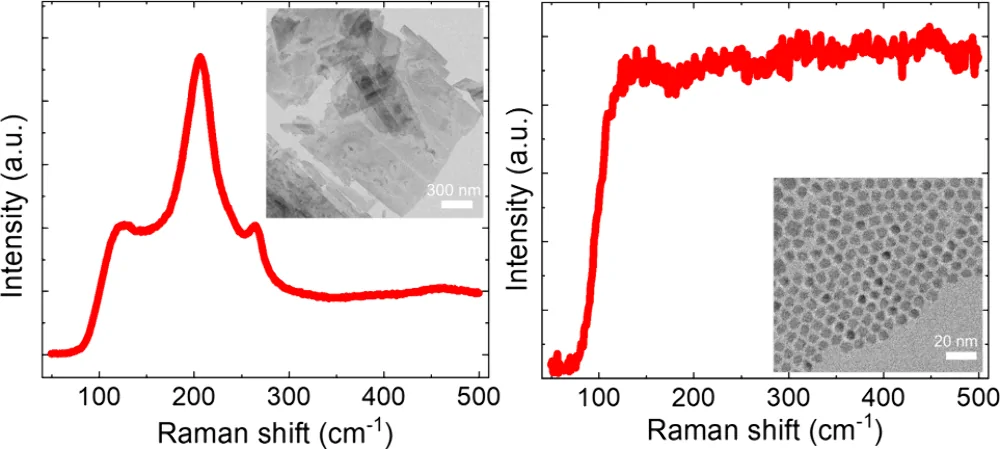

Colloidal two-dimensional (2D) semiconductor nanosheets (also known as nanoplatelets, nanoribbons, or quantum disks) have demonstrated exceptional photonic properties compared to their zero-dimensional (0D) counterparts, quantum dots (QDs). These properties include a giant-oscillator-strength-transition,^1^ low-threshold lasing,^2–4^ and highly efficient multiple-exciton generation.^5–7^ These phenomena stem from unique 2D exciton dynamics, such as suppressed Auger recombination^8,9^ and a large coherence area.^1^ These dynamics are strongly modulated by phonons confined in the 2D lattice, as evidenced by hot-phonon bottlenecks,^10,11^ and a long exciton dephasing time.^12^ In narrow-bandgap colloidal 2D nanocrystals like lead sulfide (PbS), excitons are particularly sensitive to phonon interactions at room temperature. Probing phonon physics in these systems is therefore essential for understanding exciton polarons^13,14^ and phonon polaritons,^15^ which will ultimately enable their utilization in quantum technologies.

In this Letter, we report a remarkably enhanced room-temperature Raman signal in 2D colloidal PbS nanosheets. Specifically, the longitudinal optical (LO) phonon intensity in 2D nanosheets is increased by orders of magnitude relative to 0D quantum dots. Surface symmetry breaking in these ultrathin nanosheets relaxes dipole selection rules, enabling robust Raman scattering from the intrinsically centrosymmetric PbS lattice. We show that a uniform 2D structure is essential for this enhancement, yielding a sharp, well-defined LO peak; conversely, porous nanosheets or quantum dot assemblies display weak, significantly broadened features.

Colloidal PbS nanocrystals were synthesized via wet-chemical methods (see Supporting Information A for complete synthetic details). The resulting 2D nanosheets exhibit lateral dimensions of several hundred nanometers (Figure 1c) and a thickness of a few nanometers,^16^ establishing strong quantum confinement along the thickness axis while maintaining free-carrier motion within the 2D plane. For comparison, as-synthesized 0D quantum dots possess an average diameter of 4.9 nm (Figure 1d) measured in a calibrated transmission electron microscopy (TEM) image (Supporting Information B). Samples were prepared for characterization by drop-casting the colloidal nanosheet suspension onto clean glass substrates. Following solvent evaporation, the resulting thin films were studied via Raman spectroscopy using a 785 nm excitation laser at 5 mW (Supporting Information C).

**Figure 1:**
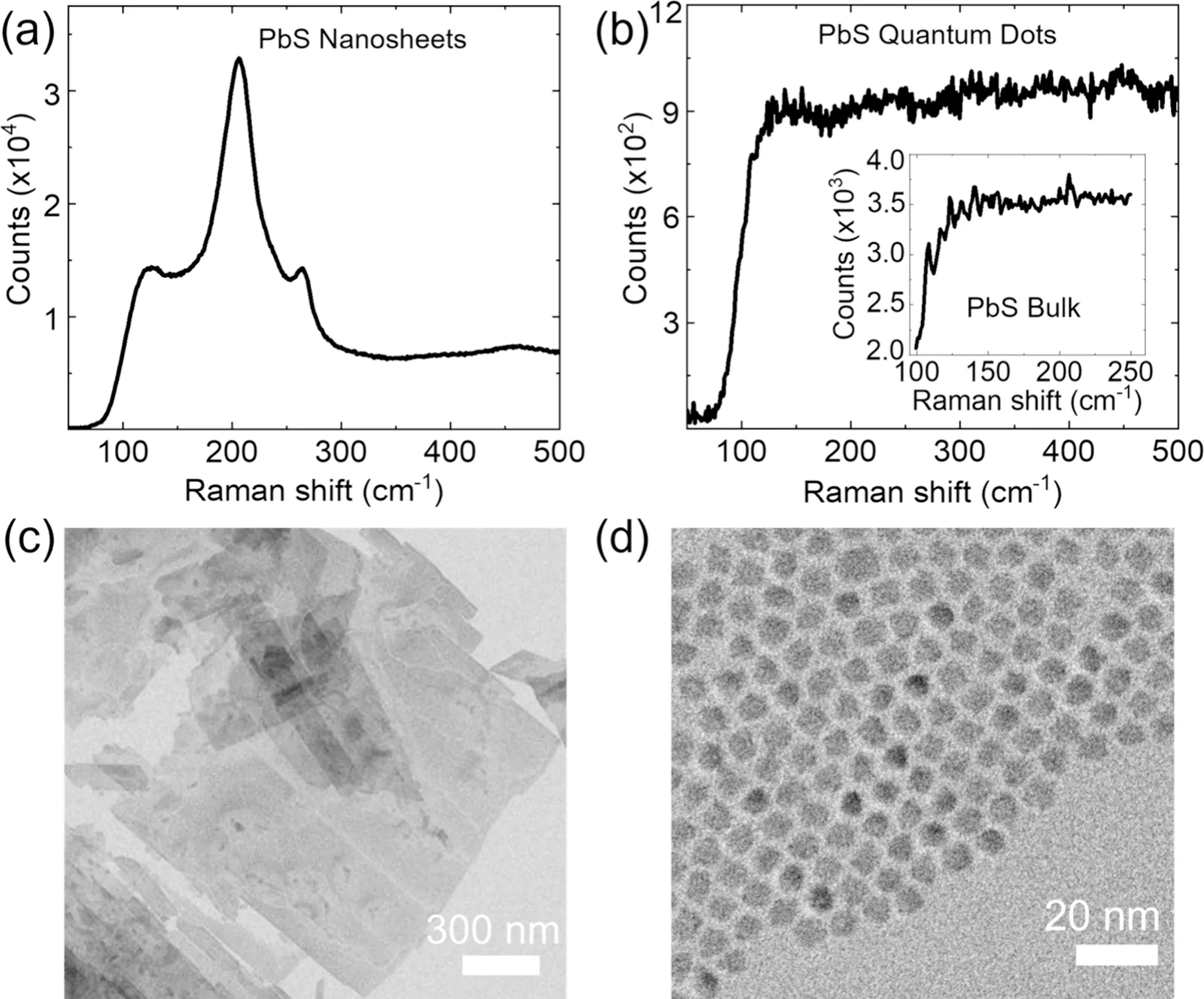
Room-temperature Raman spectra of (a) colloidal 2D PbS nanosheets and (b) 0D PbS quantum dots (inset: bulk galena-phase PbS reference). The nanosheets exhibit an intense, sharp LO phonon peak at 206 cm^–1^, which is barely resolvable in pristine quantum dots and bulk PbS. TEM images of (c) colloidal 2D PbS nanosheets and (d) ∼4.9 nm 0D PbS quantum dots.

The room-temperature Raman spectrum of the 2D nanosheets reveals a sharp principal peak at 206 cm^–1^, accompanied by a shoulder feature at 264 cm^–1^ and a broad band centered near 455 cm^–1^ (Figure 1a). The dominant peak at 206 cm^–1^ and the high-frequency band at 455 cm^–1^ correspond to the fundamental longitudinal optical (LO) phonon mode and its first overtone (2LO) near the Γ-point of the rock-salt PbS lattice.^17^ The weaker feature at 264 cm^–1^ is assigned to a two-phonon scattering process involving the overtone of the local Pb–O–Pb stretching mode, consistent with prior reports on oxidized lead chalcogenide nanostructures.^18,19^

In contrast to the nanosheets, the Raman spectrum of typical colloidal 0D PbS quantum dots barely exhibits any distinct phonon modes at room temperature (Figure 1b). Possessing a centrosymmetric rock-salt crystal structure, ideal bulk PbS is first-order Raman-inactive; even under resonant conditions, bulk galena yields only a weak room-temperature Raman response (Figure 1b, inset).^17^ Although symmetry breaking at the nanocrystal surface relaxes these selection rules to allow Raman activity, the small scattering volume and size dispersity of 0D quantum dots typically result in low-amplitude, heavily broadened peaks.^20,21^ Consequently, well-resolved Raman spectra from 0D PbS are generally restricted to cryogenic temperatures or tight resonance alignment with the lowest exciton transition.^22–24^

Despite the inherently weak scattering observed in both 0D quantum dots and 3D bulk crystals, the 2D nanosheets display an intense, remarkably sharp LO phonon peak. We attribute this dramatic enhancement to the synergistic effects of their 2D spatial geometry and 2D electronic band structure. Geometrically, collective lattice vibrations extending across the large lateral dimensions of the nanosheet can induce spatially coherent phonon coupling, thereby amplifying the macroscopic Raman cross-section. Simultaneously, the electronic confinement within the 2D morphology establishes a step-like density of states (DOS)^25^ that significantly strengthens exciton–phonon coupling.^26,27^

Optical absorption spectroscopy (Supporting Information D) confirms this step-like electronic DOS in the 2D nanosheets,^25,28^ contrasting sharply with the discrete, atomic-like absorption features of 0D quantum dots (Figure 2). While the discrete electronic spectrum of quantum dots restricts effective resonance enhancement to narrow excitation windows near the band-edge exciton absorption,^29^ the continuous, step-like DOS of 2D nanosheets ensures broad, robust resonance matching across a wide range of excitation wavelengths.^25,28^ Furthermore, the larger individual volume of a 2D nanosheet provides a substantially higher DOS relative to a single quantum dot, further enhancing the Raman signal.^27^

**Figure 2:**
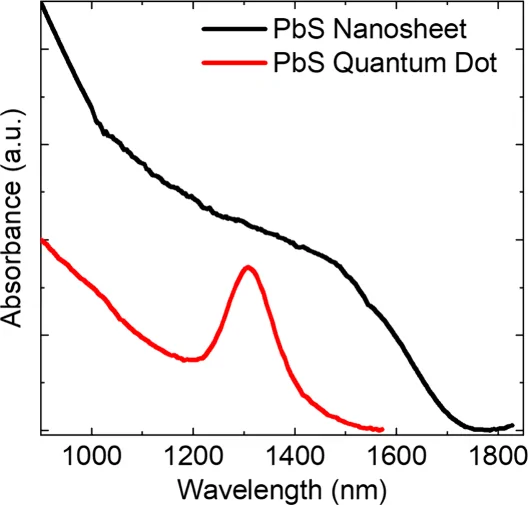
Optical absorption spectra of colloidal 2D PbS nanosheets (black) and 0D PbS quantum dots (red). The nanosheets display a step-like density of states characteristic of 2D quantum confinement, contrasting with the discrete, atomic-like exciton absorption features of 0D quantum dots.

Beyond electronic resonance enhancement, the 2D morphology directly promotes collective lattice vibrations that amplify the Raman signal. The uniform thickness^16,30^ and large lateral area facilitate spatially coherent phonon coupling, reinforcing single-frequency vibrational modes and yielding sharp, well-defined Raman peaks. In contrast, phonon coupling in 0D quantum dots is severely constrained by their limited volume. Furthermore, quantum dot films exhibit random crystallographic orientations, as evidenced by comparable X-ray diffraction (XRD) (Supporting Information E) peak intensities across multiple crystal planes (Figure 3b). Conversely, thin films of 2D nanosheets display a strikingly dominant (200) reflection (Figure 3a), confirming that the basal planes^31,32^ align preferentially parallel to the substrate surface. This combination of intra-sheet spatial phonon coherence and macroscopic texture (out-of-plane preferential orientation) dramatically enhances the total Raman scattering cross-section.

**Figure 3:**
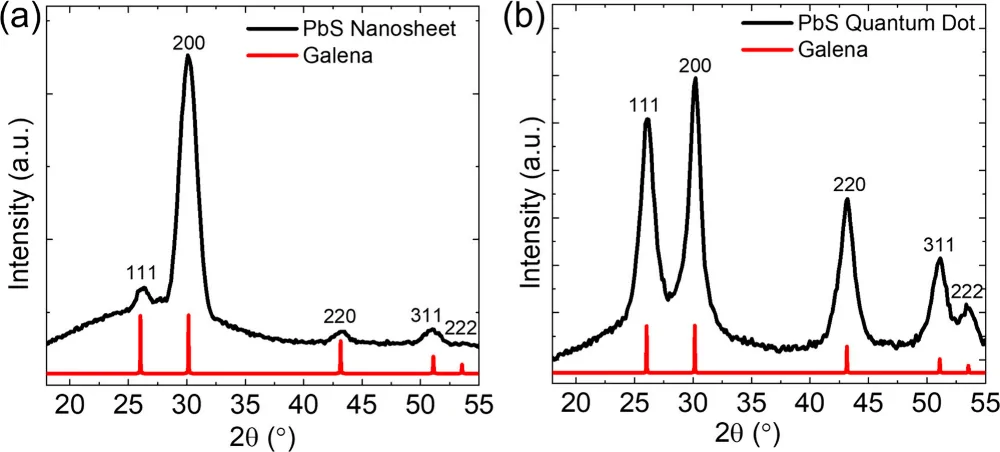
X-ray diffraction (XRD) patterns of (a) colloidal PbS nanosheets and (b) PbS quantum dots. Vertical bars at the bottom denote reference peak positions for bulk galena-phase PbS. Numbers above the diffraction peaks correspond to the respective Miller indices (*hkl*).

The crucial role of structural coherence in enhancing the Raman signal is further confirmed by tracking the morphological evolution from 0D quantum dots to porous 2D networks, and ultimately to uniform 2D nanosheets during crystal growth.^33^ Colloidal PbS nanosheets grow from quantum dots via oriented attachment.^33,34^ By isolating reaction intermediates at different growth stages, we systematically trace the transition from isolated QDs to porous 2D QD networks and, finally, to fully fused nanosheets. The intermediate porous nanosheets (Figure 4b) exhibit a weak broadened Raman peak centered at 193 cm^–1^, representing a pronounced redshift relative to the intrinsic LO phonon mode of uniform nanosheets (Figure 4a). We attribute this redshift to the dominance of surface phonon modes, which are activated by the high surface-area-to-volume ratio in porous structures.^23^ Upon ambient aging, the porous networks undergo structural coalescence^35,36^ into continuous, uniform nanosheets. Concurrently, the Raman response sharpens and intensifies dramatically (Figure 4a), converging toward the spectral signature of fully grown nanosheets (Figure 1a). While high surface porosity selectively enhances surface-phonon scattering,^37^ the continuous 2D lattice structure restores and amplifies the intrinsic bulk LO phonon mode. Taken together, this evolution—from a negligible signal in 0D QDs to a broad, redshifted feature in porous networks, and ultimately to a sharp, intense peak in solid nanosheets—directly reflects the onset of long-range structural coherence.

**Figure 4:**
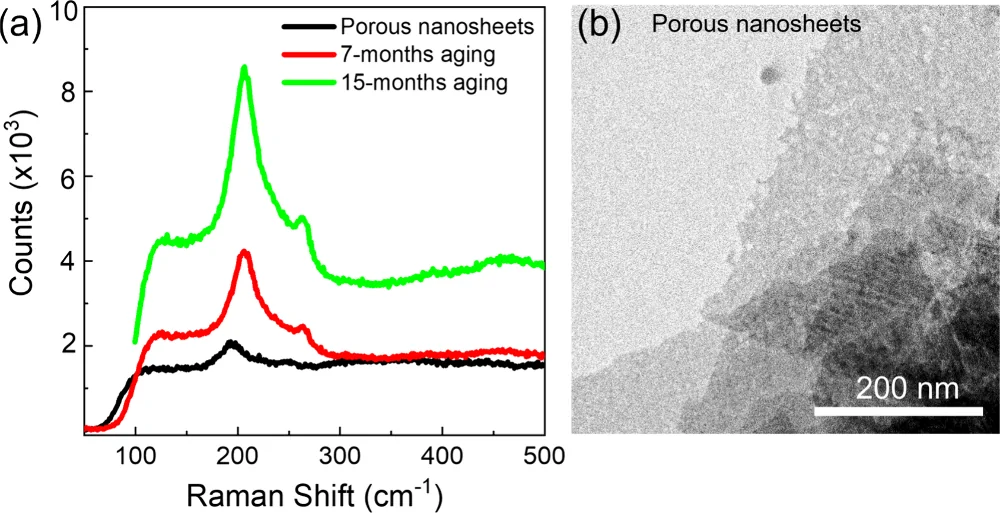
(a) Room-temperature Raman spectra of porous PbS nanosheets as-synthesized (black) and after aging for 7 months (red) and 15 months (green). The broad surface phonon peak at 193 cm^–1^ progressively shifts to 206 cm^–1^ as structural coalescence occurs. (b) Representative TEM image displaying the porous, networked morphology of nanosheet intermediates extracted during colloidal synthesis.

Although typical colloidal PbS quantum dots exhibit a weak room-temperature Raman response, the signal can be improved by modulating their surface chemistry. Specifically, oxidized PbS quantum dots display a distinct LO phonon peak at 218 cm^–1^ (Figure 5a). This feature is blue-shifted relative to the 206 cm^–1^ LO peak of 2D nanosheets, yet closely matches previously reported values for PbS quantum dots (217 cm^–1^).^23^ Storage of synthesized quantum dots in hexane rather than toluene accelerates surface oxidation due to the substantially higher oxygen solubility in hexane.^38^ XRD of these oxidized QDs reveals a prominent peak at 27.8°, assigned to the (210) reflection of lead sulfate (PbSO_4_, *a* = 8.45 Å, *b* = 5.38 Å, *c* = 6.93 Å), alongside weaker shoulder peaks at 21.2° and 33.5° (Figure 5b).^39,40^ These reflections are absent in pristine PbS QDs (Figure 3b) and uniquely confirm the formation of PbSO_4_.^41^ The resulting oxidation products (PbO and PbSO_4_) effectively activate the Raman response.^19^ We propose two complementary mechanisms for this enhancement: First, the lattice mismatch at the PbS/PbSO_4_ heterojunction breaks local inversion symmetry, relaxing selection rules to activate the intrinsically Raman-forbidden LO phonon mode of centrosymmetric PbS.^17,42^ Second, the growth of a wide-bandgap insulator (PbSO_4_) on the narrow-bandgap PbS core induces surface band bending; the resulting built-in electric field drives electric-field-induced Raman scattering,^42,43^ further amplifying the signal.

**Figure 5:**
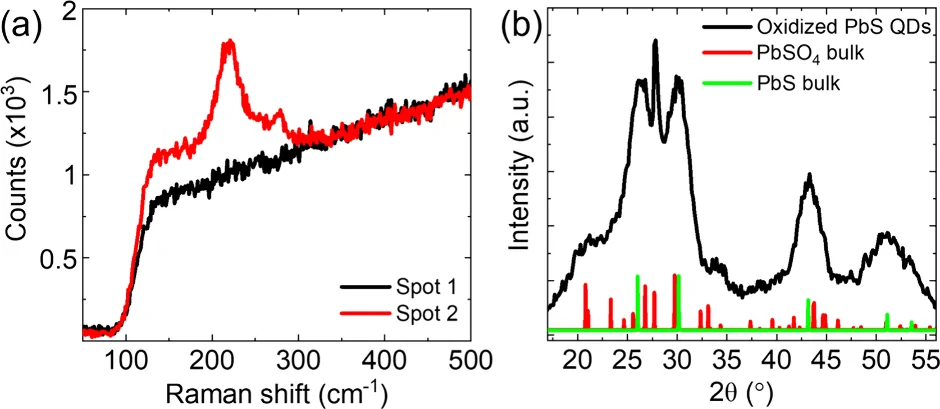
(a) Spatially resolved room-temperature Raman spectra collected at different spots on a film of colloidal 0D PbS quantum dots after extended storage in hexane (>3 years). Varied peak profiles across sampling locations demonstrate spatially non-uniform surface oxidation. (b) Powder XRD pattern of oxidized PbS quantum dots (black) referenced against standard line spectra for bulk galena-phase PbS (green) and anglesite PbSO_4_ (red). The primary QD reflections at 26.1°, 30.2°, 43.3°, and 51.1° correspond to rock-salt PbS, while the prominent peak at 27.8° confirms the formation of crystalline PbSO_4_.

The LO phonon mode in 0D PbS quantum dots exhibits a notable blue-shift relative to that of bulk PbS. This higher-frequency shift stems from the relaxation of the *q* = 0 selection rule^44^ under strong three-dimensional (3D) quantum confinement, coupled with the anomalous optical phonon dispersion of rock-salt PbS near the Γ-point of the Brillouin zone.^23,45,46^ In comparison, the LO phonon frequency of 2D nanosheets (206 cm^–1^) displays only a slight blue-shift relative to PbS bulk galena (205 cm^–1^),^17^ reflecting relaxed quantum confinement along the unconfined 2D lateral dimensions.

Although the spectral shift between nanosheets and bulk PbS is minimal, the LO Raman peak intensity from 2D nanosheets remains more than an order of magnitude higher than that of oxidation-activated 0D quantum dots (Supporting Information F). Considering the packing fraction, the absorption of the excitation light and Raman escaping light, and the thickness of the film, the experimentally obtained Raman intensity is proportional to the Raman scattering coefficient per unit volume of PbS (Supporting Information G). This analysis rules out the effect of excitation volume and demonstrates that the enhancement originates from the 2D structure.

A trace amount of surface oxide may be present on the nanosheets, as suggested by the weak Pb–O–Pb vibrational overtone at 264 cm^–1^ (Figure 1a).^19^ This overtone exists in both freshly prepared and aged nanosheets. However, X-ray diffraction patterns of the nanosheets exhibit no detectable crystalline PbSO_4_ or PbO phases (Figure 3a). The PbO layer on nanosheets likely forms a passivation layer instead of a crystal shell. This phase purity is driven by the enhanced intrinsic chemical stability of 2D nanostructures^47,48^ and the use of toluene, which features a lower oxygen solubility. Therefore, the Raman enhancement in 2D nanosheets is unlikely to be from surface oxidation artifacts. The symmetry breaking along the surface normal of the (200) basal plane and the preferential orientation of large (200) facets of the nanosheets are likely the origins of the massive Raman enhancement.

In conclusion, we have demonstrated a remarkable, order-of-magnitude enhancement of room-temperature Raman LO phonon modes in colloidal 2D PbS nanosheets relative to 0D quantum dots and 3D bulk galena. We attribute this dramatic amplification to the interplay of spatial phonon coherence across the expansive 2D lattice and resonant coupling facilitated by the step-like electronic density of states. Unlocking this structurally driven, intrinsic Raman signal under ambient conditions provides a non-destructive optical framework to directly probe lattice dynamics in colloidal 2D nanomaterials. These insights into 2D phonon physics are vital for unraveling the exciton–phonon interactions that govern hot-carrier cooling, multiple exciton generation, and superfluorescence in next-generation optoelectronic and quantum devices.

## Supplementary Material



## Notes

Synthesis, transmission electron microscopy, Raman spectroscopy, optical spectroscopy, X-ray diffraction, Raman intensity comparison, and Raman scattering coefficient estimation (PDF)
