# Supplementary material for: Room-Temperature Raman Enhancement in Colloidal 2D PbS Nanosheets
Source: J Phys Chem Lett. 2026 Sep 2;17(37):10697–701. doi: 10.1021/acs.jpclett.6c02525 (PMC13592083; doi:10.1021/acs.jpclett.6c02525)
Supplement: Supplementary file 1 [file jz6c02525_si_001.pdf]

Supporting Information

Room-Temperature Raman Enhancement in Colloidal 2D PbS Nanosheets

Sabin Aryal,[1,2] Yiteng Tang,[1,2] and Liangfeng Sun[1,2*]

[1]Department of Physics and Astronomy, Bowling Green State University, Bowling Green, Ohio 43403, USA

[2]Center for Photochemical Sciences, Bowling Green State University, Bowling Green, Ohio 43403, USA

A. Synthesis

Synthesis of lead sulfide nanosheets. The lead precursor was synthesized by dissolving lead oxide (PbO, 506 mg, 99%, Sigma-Aldrich) in a mixture of diphenyl ether (10 mL, 99%, Thermo Scientific) and oleic acid (1.8 mL, 90%, Aldrich) inside a three-neck flask. The mixture was initially heated to 110 ˚C and degassed at 100 ˚C under vacuum. Following degassing, the reaction mixture was re-heated to 110 ˚C, and 1,1,2-trichloroethane (1 mL, 98%, Thermo Fisher Scientific) was injected under a nitrogen atmosphere. The mixture was allowed to react for 30 min. Separately, the sulfur precursor was prepared by dissolving thioacetamide (TAA, 12 mg) in a co-solvent mixture of *N*,*N*-dimethylformamide (70 µL, 99.8%, Thermo

Fisher Scientific) and trioctylphosphine (930 μL, 90%, Sigma-Aldrich) under continuous stirring in a nitrogen atmosphere.

Upon reaching the target reaction temperature, the sulfur precursor solution was rapidly injected into the vigorously stirred lead precursor solution. The reaction was maintained at this temperature for a specified duration to control nanosheet growth before cooling naturally to room temperature. The resulting colloidal product was purified by washing three times with toluene and isolating the precipitate via centrifugation. Finally, the purified PbS nanosheets were redispersed in toluene and stored under ambient conditions.

To isolate intermediate porous nanosheets, an aliquot of the reaction mixture was extracted after 5 min of growth and rapidly quenched in an ice bath to arrest further reaction.

<u>PbS Quantum dot synthesis.</u> In a typical synthesis, lead(II) oxide (PbO, 0.220 g, 99%, Sigma-Aldrich) was combined with oleic acid (OA, 0.64 mL, 90%, Aldrich) and 1-octadecene (ODE, 9.96 mL, 90%, Sigma-Aldrich) in a three-neck flask. The mixture was heated to 150 ˚C with continuous stirring under a nitrogen atmosphere for 1 h to form the lead oleate precursor, after which the solution was adjusted to the target reaction temperature. Separately, the sulfur precursor was prepared by mixing 1-octadecene (6 mL) and hexamethyldisilathiane (HMDS, 126 μL, synthesis grade) in a flask, followed by stirring for 30 min under nitrogen. Once the lead precursor reached the desired temperature, the sulfur precursor was rapidly injected. The reaction was allowed to proceed for 1–5 min (depending on the target particle size and temperature) before being quenched in an ice bath. The resulting quantum dots were purified

by precipitation with a methanol/butanol mixture, collected via centrifugation (4500 rpm, 5 min, repeated twice), and finally redispersed in toluene for storage.

B. Transmission electron microscopy

Transmission electron microscopy (TEM) images were acquired on a Hitachi HD-2300 scanning transmission electron microscope (STEM). TEM samples were prepared by drop-casting 10 μL of colloidal PbS nanosheet or quantum dot suspension onto a carbon-coated copper grid (300 mesh, Ted Pella, Inc., No. 01840-F) and drying overnight under vacuum. Particle size distributions for the quantum dots were determined by measuring the average core diameter across n = 40 individual nanocrystals from TEM images using ImageJ software (Figure S1).

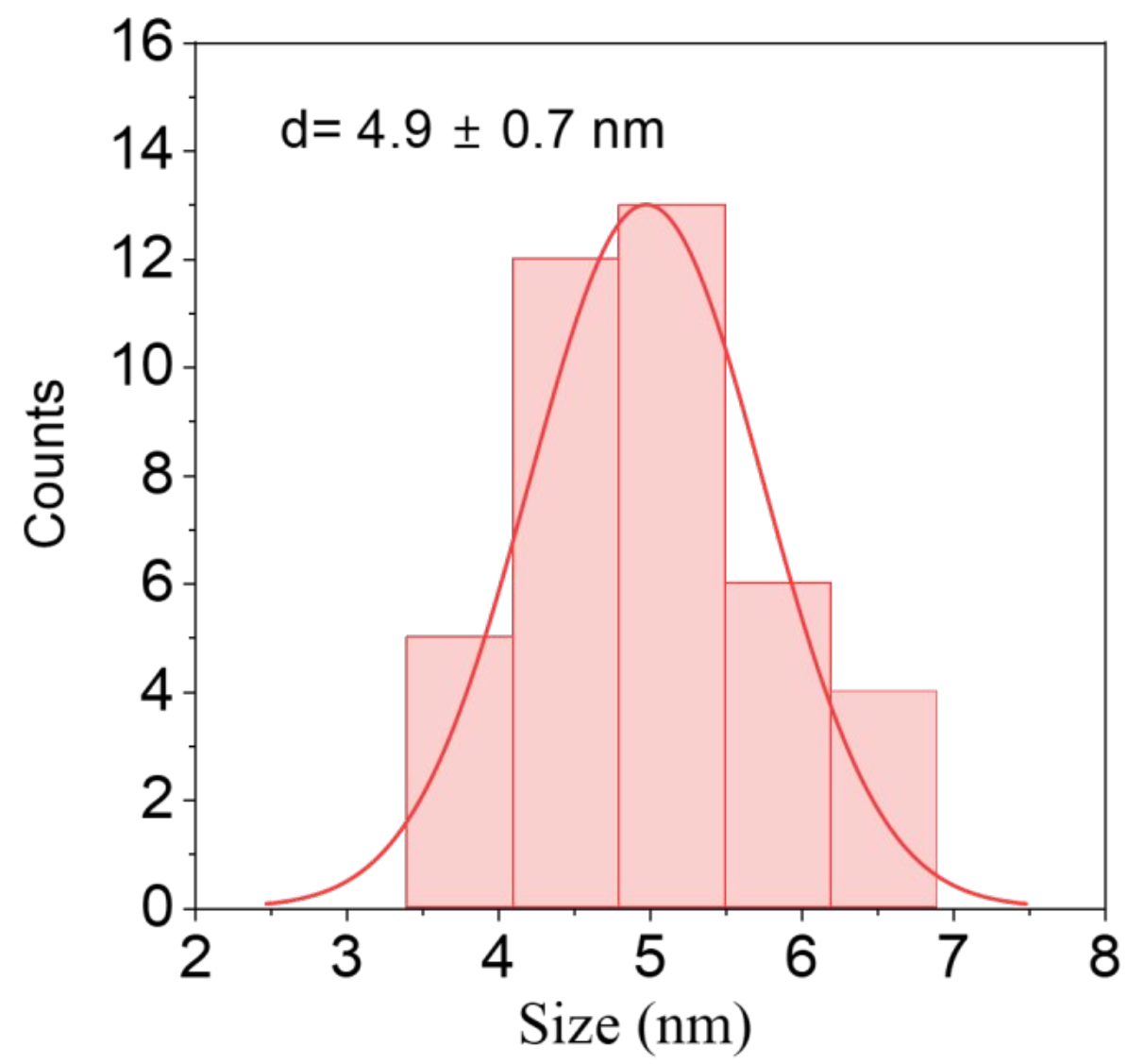

Figure S1. Histogram of the diameter of quantum dots. Gaussian fitting shows an average diameter of 4.9 nm and a standard deviation of 0.7 nm.

C. Raman Spectroscopy

Room-temperature Raman spectra were acquired using a Renishaw inVia Raman microscope equipped with an integrated 785 nm diode laser and a high-sensitivity Renishaw CCD detector. Incident laser excitation was focused onto the sample, and backscattered light was collected through a 5x objective lens (NA = 0.12) using a 1200 lines/mm diffraction grating. Wavelength calibration was validated against the longitudinal optical phonon mode of a standard silicon reference substrate at 520.7 cm^{-1}. Instrument operation, data acquisition, and baseline processing were performed using WiRE 3.4 software. To ensure quantitative reproducibility,

all spectra were collected under identical laser power density, integration time, and optical alignment, unless explicitly noted otherwise.

Microscope glass substrates were sequentially cleaned with methanol and sonicated in toluene prior to sample deposition. For film preparation, 1 mL of colloidal nanostructure suspension (2 mg/mL) was concentrated under a nitrogen stream, resuspended in 0.5 mL of fresh toluene, and briefly vortexed. The resulting suspension was drop-cast onto the cleaned glass substrate and dried under ambient conditions. This drop-casting cycle was repeated sequentially to yield a uniform, solvent-free film of optimal thickness for optical probing.

D. Optical Spectroscopy

Optical absorption measurements are performed using a home-built system. It includes a quartz tungsten-halogen lamp (Oriel 66187), an integrating sphere (Thorlabs IS200-4), a monochromator (Acton SP-2357, Princeton Instruments), and an IR femtowatt photoreceiver (Model 2153, New Focus Inc). The transmitted light was dispersed through a monochromator (Acton SP-2357, Princeton Instruments) and detected using an IR femtowatt photoreceiver (Model 2153, New Focus, Inc.). Colloidal PbS nanosheets or quantum dots were dispersed in tetrachloroethylene (TCE) and transferred to a quartz cuvette (Starna Cells, Inc.) for absorption and photoluminescence measurements.

E. X-ray diffraction

Powder X-ray diffraction (XRD) patterns were collected on a Bruker D8 Advance diffractometer using Cu tube (λ = 1.54 Å) and a high-performance LYNXEYE position-sensitive detector.

F. Raman intensity comparison

The Raman measurements on nanosheet and quantum-dot films were performed under identical optical conditions (laser wavelength, objective, excitation power, and acquisition time). The films were sufficiently thick to be optically opaque at the excitation wavelength, providing comparable optical sampling volumes. Because the amount of PbS within the illuminated volume was approximately the same, the markedly stronger LO Raman signal observed in the nanosheet films indicates a substantially larger effective Raman scattering efficiency than in the quantum-dot films.

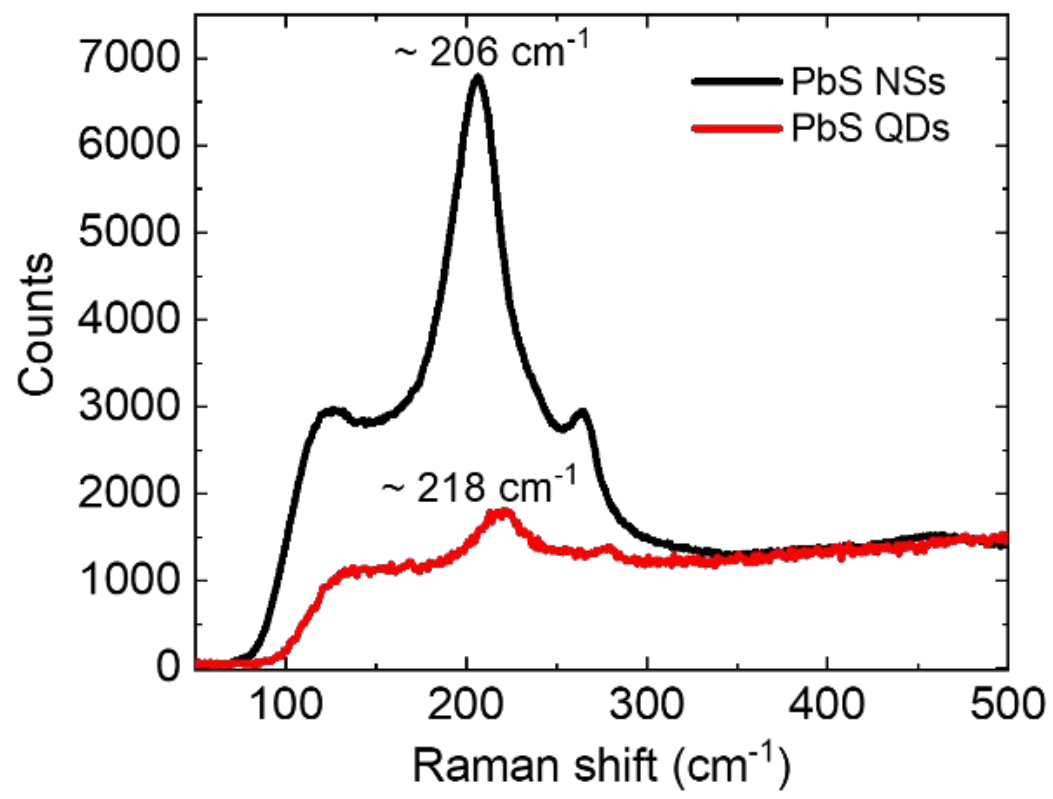

Figure S2. Raman shift for the PbS nanosheets (black solid line spectrum) and PbS quantum dots (red solid line spectrum).

G. Raman scattering coefficient estimation

In experiments, the intensity of Raman signal is proportional to the amount of PbS sampled, the Raman cross section (σ_R) and the optical weighting. Since we kept the excitation laser spot the same size (~ 8 microns) on all measurements, the amount of PbS sampled is proportional to the packing fraction and the depth of the excitation.

The packing fraction is calculated by dividing the total volume of PbS core by the total volume of the oleic acid capped PbS core. For the nanosheet film, we choose a typical nanosheet with thickness of 2 nm, width of 600 nm and length of 800 nm. The layer of oleic acid is about 1.8 nm,[1] capping the whole nanosheet. It increases each dimension by 3.6 nm. Therefore, the packing fraction

$$f_{NS} = V(core)/V(total) = (2 \times 600 \times 800)/(5.6 \times 603.6 \times 803.6) = 0.35$$

For quantum dot film, the average diameter of the PbS core is 4.9 nm. The oleic acid layer increases the total diameter to 8.5 nm. Therefore, the packing fraction

$$f_{QD} = V(core)/V(total) = 4.9^3/8.5^3 = 0.19$$

We make the film thick enough so that no laser light can pass through it. In such cases, the depth of the excitation is determined by the effective absorption coefficient of the film. Taking into account the attenuation of the Raman photons while escaping the film, the Raman intensity

$$I_R \propto f S_R I_0 \int_0^d e^{-\alpha_{exc} z} e^{-\alpha_R z} dz \, .$$

In the above equation, S_R is the Raman scattering coefficient per unit volume of PbS, f is the PbS volume fraction in the film, I_0 is the excitation laser intensity. α_{exc} and α_R are the absorption coefficients of film at excitation wavelength and Raman wavelength, respectively. Since oleic acid is transparent at the excitation wavelength, they are related to the absorption coefficients of PbS via PbS volume fraction (f): $\alpha_{exc} = f\alpha_{PbS,exc}$ and $\alpha_R = f\alpha_{PbS,R}$. Given that, the Raman intensity

$$I_R \propto f S_R I_0 \frac{1 - e^{-f(\alpha_{PbS,exc} + \alpha_{PbS,R})d}}{\alpha_{PbS,exc} + \alpha_{PbS,R}} \, .$$

Since the excitation wavelength and the Raman wavelength are close to each other, we can set $\alpha_{PbS,R} = \alpha_{PbS,exc} = \alpha_{PbS}$. Also, the thickness of the film d is much larger than the attenuation length of the excitation, the exponential term in the last equation is ~ 0. So, the Raman intensity in our setup is proportional to $S_R f_{PbS}/\alpha$. Consider $\alpha = f_{PbS}\alpha_{PbS}$, the intensity is then proportional

to S_R/α_{PbS}. Since the excitation wavelength is off the resonance of NSs and QDs, α_{PbS} is roughly the same for both. Therefore, the experimentally obtained Raman intensity is proportional to S_R.

References:

(1) Schliehe, C.; Juarez, B. H.; Pelletier, M.; Jander, S.; Greshnykh, D.; Nagel, M.; Meyer, A.; Foerster, S.; Kornowski, A.; Klinke, C.; Weller, H. Ultrathin PbS Sheets by Two-Dimensional Oriented Attachment. Science 2010, 329 (5991), 550–553. https://doi.org/10.1126/science.1188035;
